# Integrated study on the comprehensive magnetic-field configuration performance in the 150 kW superconducting magnetoplasmadynamic thruster

**DOI:** 10.1038/s41598-021-00308-4

**Published:** 2021-10-19

**Authors:** Jinxing Zheng, Haiyang Liu, Yuntao Song, Cheng Zhou, Yong Li, Ming Li, Haibin Tang, Ge Wang, Yuntian Cong, Baojun Wang, Yibai Wang, Peng Wu, Timing Qu, Xiaoliang Zhu, Lei Zhu, Fei Liu, Yuan Cheng, Boqiang Zhao

**Affiliations:** 1 Institute of Plasma Physics, Hefei Institutes of Physical Science, Chinese Academy of Sciences, Hefei, 230031 China; 2 University of Science and Technology of China, Hefei, 230026 China; 3 Beijing Institute of Control Engineering, Beijing, 100080 China; 4 Beihang University, Beijing, 100191 China; 5 Tsinghua University, Beijing, 100084 China

**Keywords:** Space physics, Engineering, Physics

## Abstract

Higher magnetic fields are always favoured in the magnetoplasmadynamic thruster (MPDT) due to its superior control of the plasma profile and acceleration process. This paper introduces the world's first integrated study on the 150 kW level AF-MPDT equipped with a superconductive coil. A completely new way of using superconducting magnet technology to confine plasma with high energy and extremely high temperatures is proposed. Using the PIC method of microscopic particle simulation, the plasma magnetic nozzle effect and performance of the MPDT under different magnetic-field conditions were studied. The integrated experiment used demonstrated that, in conjunction with the superconducting coil, greater homogeneity and a stronger magnetic field not only caused more even cathode ablation and improved its lifespan but also improved the performance of the MPDT (maximum thrust was 4 N at 150 kW, 0.56 T). Maximum thrust efficiency reached 76.6% and the specific impulse reached 5714 s.

## Introduction

The high (specific) impulse, high thrust and longer operational life of the high-power magnetoplasmadynamic thruster (MPDT)^1,2^ make it a potential electric thruster for various space applications, from performing orbital manoeuvres to interplanetary missions^3–7^. The profile of the plasma is controlled by the magnetic field and electric field, and then it is rejected to produce thrust. This means the interaction between the magnetic field and the plasma plays a significant role in improvements to impulse and thrust. The applied field-type MPDT (AF-MPDT), with its high efficiency and specific impulse, has been receiving increasing attention due to its higher magnetic field. Traditional space propulsion technology, such as chemical propulsion, is limited by its reliance on propellant energy. It consumes a considerable amount of propellant over a short time and produces a lot of thrust, but the specific impulse remains small, and is nearly 10 times lower than that of an electric propulsion system consuming the same mass of propellant. Developments in conventional electric propulsion technology have been limited due to the large magnet and small magnetic fields involved, as well as electrode corrosion and sputtering, both of which are difficult to resolve to further improve the performance of the thruster^8^.

Many research institutions have studied on SF- MPDT or AF-MPDT systems with > 100 kW of power. For example, NASA's Lewis Research Center conducted a performance test of an AF-MPDT with a power level of approximately 100 kW, applied magnetic field intensity of 0.15 T, thrust of 2.23 N, specific impulse of 2280 s, and efficiency of 22.8%^9,10^. The 20–250 kW AF-MPDT designed by Alta S.P.A. (Italy) has an applied magnetic field strength of 0.12 T, a thrust efficiency of 22% at 100 kW, and a specific impulse of 2500 s^11^. The SX3 thruster designed by Institute of Space Systems (IRS), University of Stuttgart in Germany reaches a power of approximately 100 kW, an applied magnetic field strength of 0.4 T, and an efficiency of 60%^12–14^. However, the maximum magnetic field of the applied magnet generally does not exceed 0.4 T. James. S. Sovey of the Lewis Research Center revealed that "by changing from purely self-field to applied magnetic field, the specific impulse of a pulsed hydrogen thruster was increased by nearly 60%”^15^, which means the intensity of the applied magnetic field represents a key parameter capable of affecting the overall performance of the thruster. Considering the strong current density inherent in superconducting technology, the weight, volume and power consumption could be reduced significantly, which would also cater to the development trend of low-cost space missions^16^. Superconducting magnets represent a feasible way to extend the operational range of MPD thrusters^17^ and enable higher discharge voltages, to a certain extent. This allows for applications that use lower discharge currents and hollow cathodes at the same electrical power level, which are favourable in ionizing propellant into plasma^14,18,19^.

**Figure 1:**
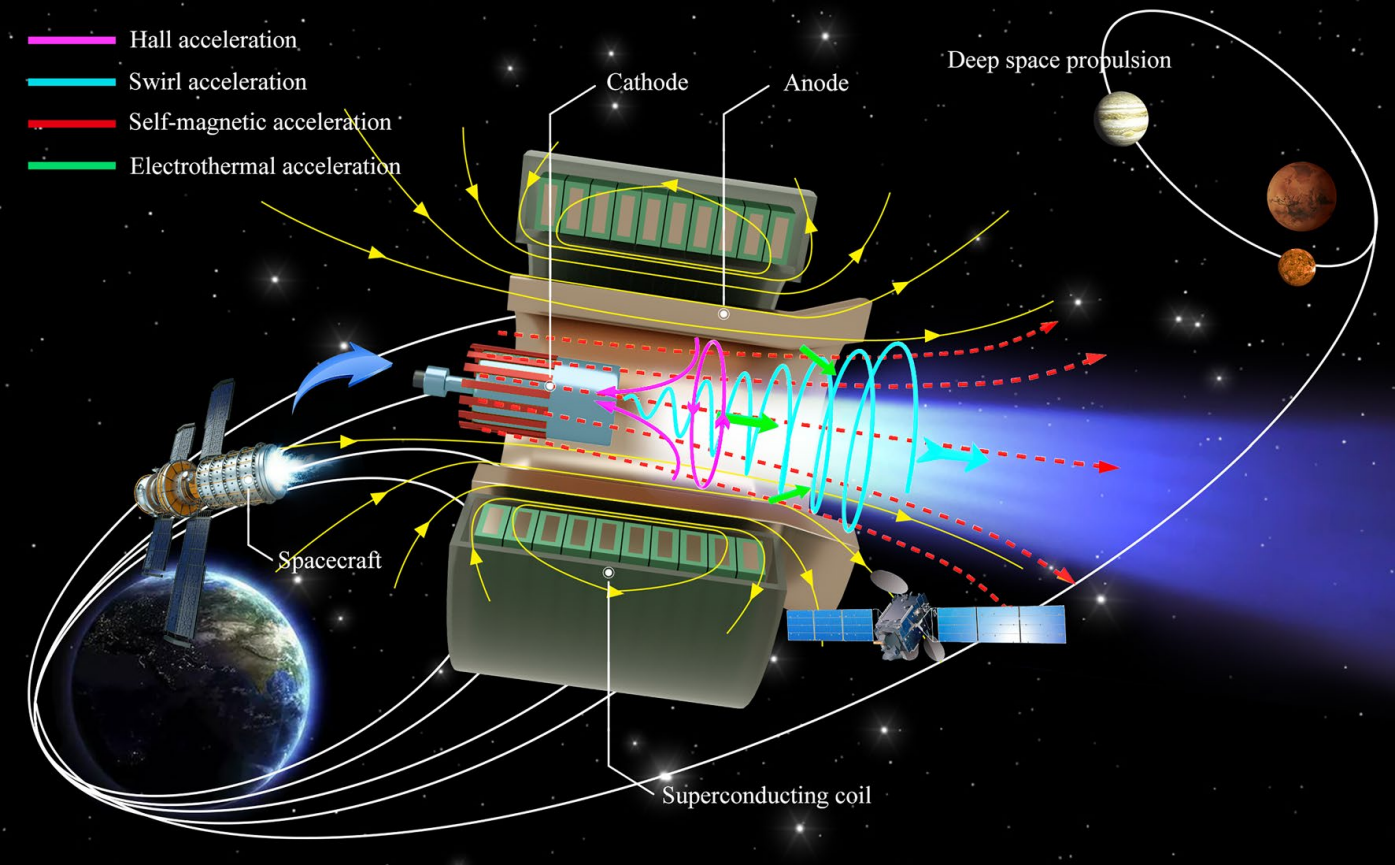
Schematic illustration of the working principle of the superconducting MPDT. The acceleration mode of the MPDT includes Hall, swirl, self-magnetic and electrothermal acceleration. The superconducting MPDT consisted predominantly of a cathode, anode and superconducting coil and the thruster is suitable for future use in LEO satellites and deep-space propulsion for its high specific impulse, high thrust and longer operational life.

To date, only ASIPP and BICE have pursued the development and performance testing of 150 kW level superconducting MPDT (AF-MPDT equipped with a superconductive coil). This paper introduces the world's first integrated 150 kW level MPDT with a superconducting magnet. The system integration and ground experiment were completed, with results revealing a 5714-s impulse, 4-N thrust and efficiency of 76.6% at a mass flow rate of 70 mg/s and power of 150 kW. The promotion of the key parameters of the MPDT substantiates notions that increasing the magnetic field can improve overall MPDT performance. The process underpinning the ignition test was very successful and the superconducting magnet performed well during plasma discharge, which attests to the feasibility of applying superconducting technology to MPDT. Compared with conventional copper coil, the central magnetic field of the superconducting coil was greatly increased, from 0.4 to 1 T, while the size and weight of the coil decreased considerably. The optimisation of the design and magnetic-field profile of the superconducting magnet used in AF-MPDT were studied with respect to the acceleration process. The acceleration process of the AF-MPDT includes four acceleration modes, as shown in Fig. 1. For plasma with a high degree of ionisation, self-magnetic acceleration, Hall acceleration and swirl acceleration constitute the most important acceleration modes^20,21^. The current studies demonstrated that superconducting magnet has a significant effect on the performance of the MPDT for its high-current density and magnetic-field uniformity.

## Results

### Device and magnetic field configuration

**Figure 2:**
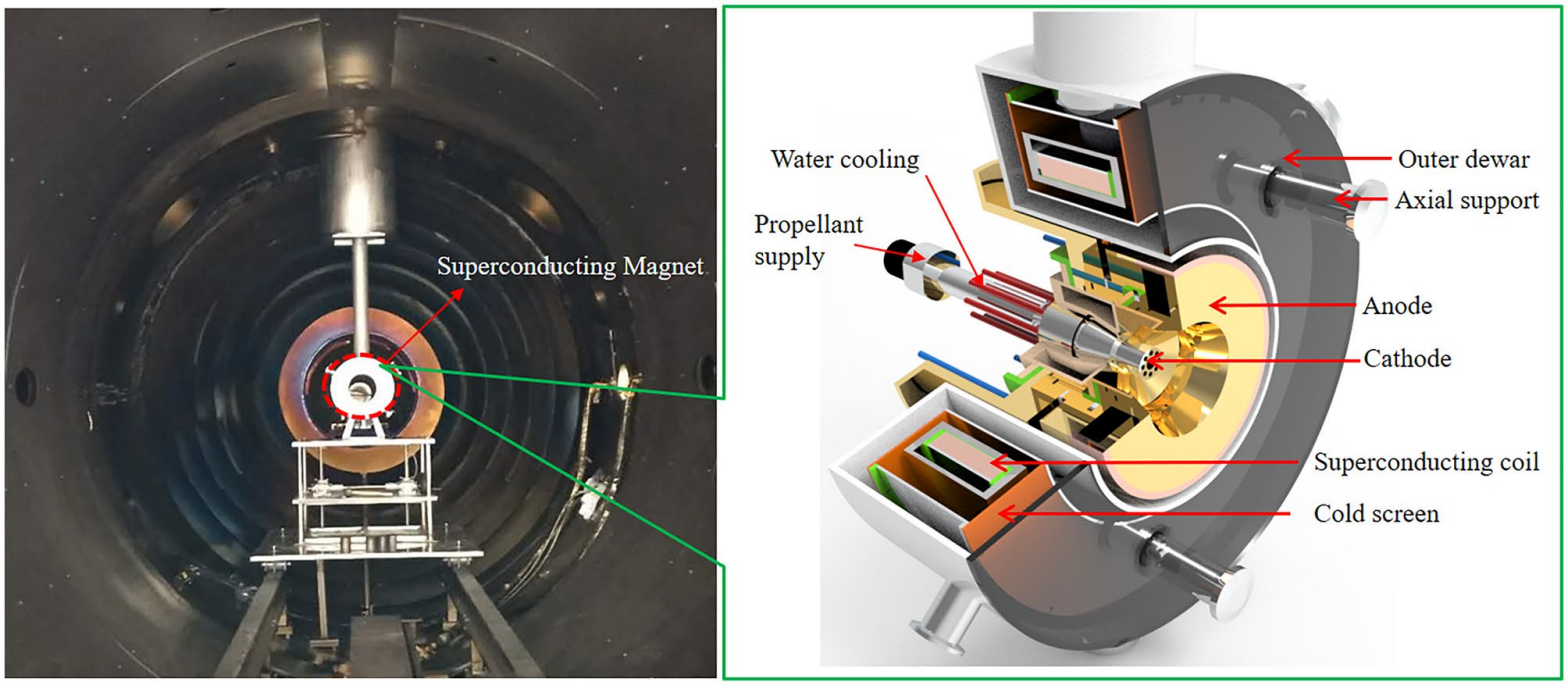
Structure of superconducting MPDT. The 150 kW superconducting versus the copper magnet in the MPDT under vacuum environment. The superconducting MPDT predominantly consists of an anode, cathode, superconducting coil, cold screen, axial support, outer dewar, propellant supply, and water cooling (among others).

The experiments in this study were conducted through a superconducting MPDT predominantly consisting of an anode, cathode, superconducting magnet, propellant (among others), as shown in Fig. 2. Common propellants for MPDT include inert gases, alkali metals, and hydrogen molecules (among others). Among these, alkali metals cause potential pollution of spacecraft^22^ and hydrogen molecules cause serious cathode ablation due to the relatively small atomic mass of dissociated and ionised products, such that more plasma is produced near the anode and cathode as confirmed by Uematsu et al.^23^. As a low-cost inert gas with low ionisation energy, argon gas is a mainstream propellant of MPDT. Moreover, at a given mass flow rate, the lower the atomic mass of propellant, the denser and more efficient the ionized plasma will be^23^. Argon gas was pumped through a mass flow controller from the aperture of the multi-cathode and then ionised to plasma at a relatively higher voltage (with a maximum rated supply voltage of 10 kV) between the anode and cathode.

The profile of the plasma was precisely controlled by the strong magnetic field. Two semi-empirical formulas that can be used to represent thrust ***F***_***T***_ can be expressed by Eqs. (1)^10^ and (2)^24^,

1 $$F_{T1} = JB_{A} \frac{{r_{a}^{2} }}{{500r_{c} l_{c} }}$$

2 $$F_{T2} = 2KJB_{A} r_{a}^{x}$$

which are based on the mathematical derivations and experimental data modelling of the MPDTs of different research institutions. Here, *J* represents the arc current between the anode and cathode; *K* is a constant; and *r*_*a*_, *r*_*c*_ and *l*_*c*_ are the radius of the anode and cathode, and the length of the cathode, respectively; *B*_*A*_ is the applied magnetic field; and *x* is a fitting coefficient. It is important to note that the arc current and the dimensions of the MPDT cannot be significantly improved to increase thrust. Based on Eqs. (1) and (2), the thrust *F*_*T*_ is in direct proportion to the applied magnetic field *B*_*A*_, which can be significantly improved using a superconducting magnet.

Moreover, the plasma profile control can also be affected by the magnetic profile. The use of a magnetic nozzle to accelerate a plasma plume involves a process in which the plasma is magnetised (or partially magnetised), which in turn leads to timely magnetisation release. The four acceleration-mode^25,26^ types can be expressed via Eqs. (3), (4), (5) and (6). Through the superposition of the four acceleration modes, plasma can be accelerated and ejected, which has great potential for application to accelerator technology^27^. Each acceleration process is closely related to the various direction components of the magnetic field. Therefore, in the acceleration channel, the magnetic field strength and magnetic potential must meet the requirements. With the carefully designed and optimised magnetic-field profile of the superconducting magnet, the requirements in terms of magnetic-field strength and magnetic potential can be met. The performance of the thruster was found to improve with the utilisation of the superconducting magnet versus the traditional copper magnet.

3 $$F_{{{\text{swirl}}}} = \mathop \int \nolimits_{V}^{ } \left( { - j_{r} B_{z} + j_{z} B_{r} } \right)dV$$

4 $$F_{{{\text{aerodynamic}}}} = \mathop \int \nolimits_{S}^{ } \left( {kn_{i} T_{i} + kn_{e} T_{e} + kn_{a} T_{a} } \right)dS$$

5 $$F_{{{\text{hall}}}} = \mathop \int \nolimits_{V}^{ } \sqrt {\left( {j_{\theta } B_{r} } \right)^{2} + \left( {j_{\theta } B_{z} } \right)^{2} } dV$$

6 $$F_{{{\text{self}}}} = \mathop \int \nolimits_{V}^{ } \sqrt {\left( {j_{r} B_{\theta } } \right)^{2} + \left( {j_{z} B_{\theta } } \right)^{2} } dV$$

Table 1 presents the specific parameters of the superconducting magnet used in the AF-MPDT. The magnetic-field lines of the superconducting solenoid were designed parallel to the inner surface of the dilated anode to achieve a superior thruster performance^28^. The central magnetic field of the superconducting coil was found to reach > 1 T, while that of the copper magnet could reach no more than 0.4 T for the common application considered in our experiment. The application of the superconducting magnet greatly reduced the size and weight of the magnet system (the outer diameter decreased from 900 to 350 mm and the weight from 150 to 75 kg), which is of great importance in outer space.

**Table 1:** Parameters of the superconducting magnet for the MPDT.

Name	Specification
Diameter of coil	240 mm
Diameter of outer Dewar	173 mm
Central magnetic field	> 1 T
Maximum magnetic field	> 2.8 T
Homogeneity error (R ≤ 20 mm)	< 1.7%
Critical current of superconducting wire 4 T (4.2 K)	> 710 A

**Figure 3:**
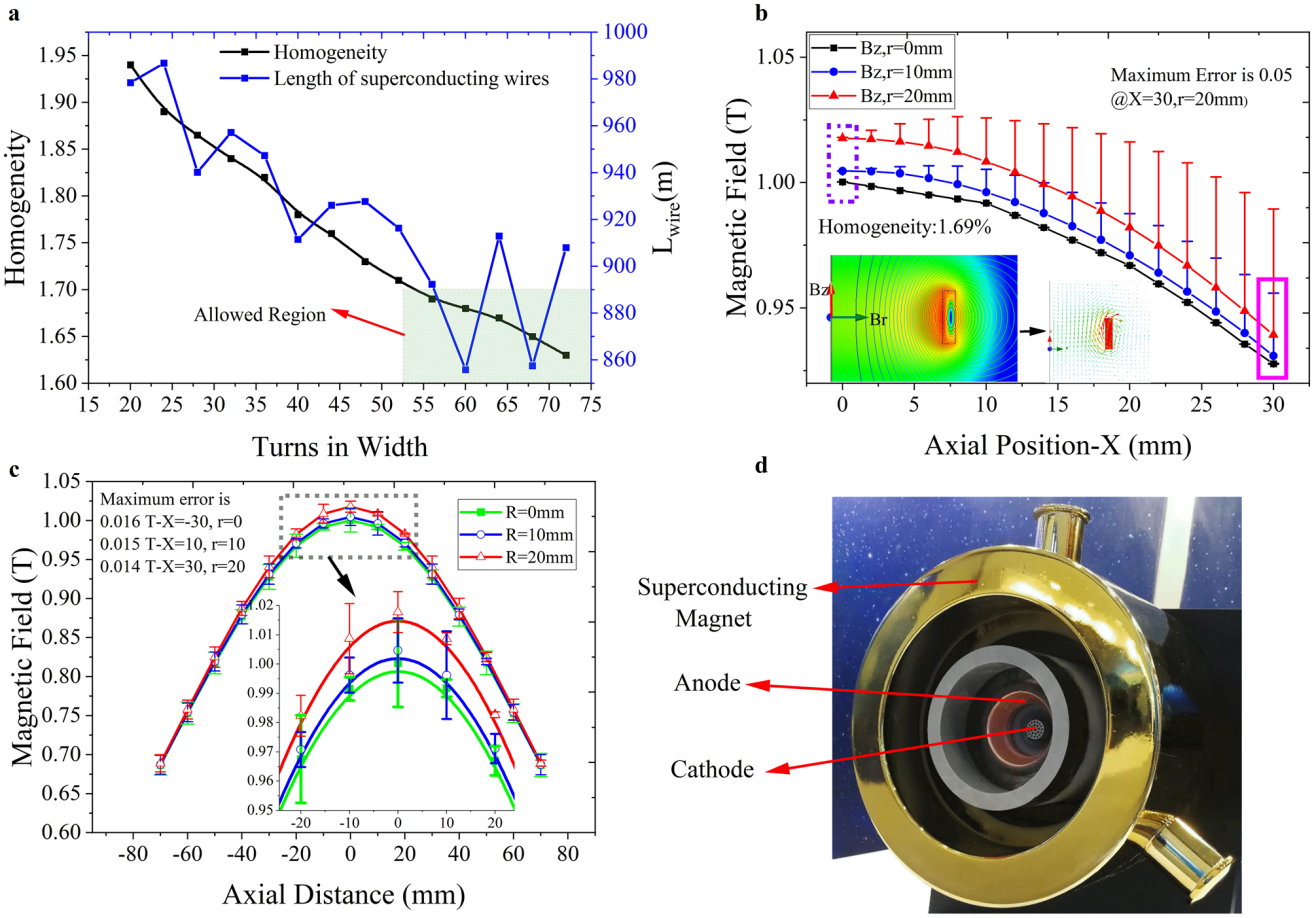
(**a**) Central magnetic field homogeneity of coil types within the radius of 20 mm and their length of wire. (**b**) Axial magnetic field component (Bz) and radial magnetic field component (Br) distribution of the superconducting magnet offset by different distances along the radial direction (r = 0 mm, r = 10 mm, r = 20 mm). (**c**) Measured magnetic field on the axial line (r = 0 mm, r = 10 mm and r = 20 mm) from the distance − 70 mm to 70 mm was compared with the analysis results, and the maximum error was 0.016 T, with a maximum error rate of 1.6%. (**d**) Schematic diagram of superconducting MPDT.

Previous studies have confirmed that slim magnetic-field configuration has a significant effect on the improvement of MPDT performance^29^. The magnetic-field distributions of several kinds of coil configurations were calculated with unchanged ampere-turns. The number of turns in the direction of width varied from 20 to 72. Figure 3a shows the relationship between the central magnetic field uniformity error within a radius of 20 mm (ε_R = 20 mm_) and the number of turns in the direction of the width. The curve indicates that the uniformity error was inversely proportional to the number of turns in this direction. The corresponding length of the whole coil also changed under the condition of the same law. Given the limitation of the uniformity error (see Table 1) and the usage of the superconducting wire, the scheme of 56 turns in the width direction of the coil with ε_R = 20 mm_ < 1.7% was adopted. Given also the limitations to the matching of the thruster and superconducting magnet, the magnetic-field line of the coil matched the expansion of the anode surface promisingly. Furthermore, the length of the superconducting wire L_wire_ was also relatively short in this case.

Hall acceleration and swirl acceleration of the plasma are predominantly produced via the applied magnetic field. Swirl acceleration plays a dominant role in the acceleration process. Swirl acceleration refers to the circumferential Lorentz force generated by the interaction of the axial and radial discharge currents (**J**_Z_, **J**_R_) with the two components of the applied magnetic field (**B**_R_, **B**_Z_). The Lorentz force (**J**_R_ × **B**_Z_), which is produced by the radial discharge current J_R_ and axial magnetic field component B_Z_, acts as a torque to transfer kinetic energy to plasma. The Lorentz force (**J**_Z_ × **B**_R_) produced by the axial current component (J_Z_) and radial magnetic field component (B_R_) can also convert part kinetic energy to the axial momentum of plasma via the use of an expanded magnetic nozzle; however, the axial magnetic field component (B_Z_) plays a leading role in this. Figure 3b shows the axial magnetic field component (B_Z_) and radial magnetic field component (B_R_) distribution of the superconducting magnet offset by different distances in the axial direction. The results indicate that the homogeneity of the magnetic field is capable of reaching 1.69% in the radial range of 20 mm for the axial line from the distance − 70 to 70 mm. Additionally, the radial magnetic field component was found to be relatively small compared with the axial magnetic field component, indicating that the magnetic-field profile of the superconducting magnet is capable of meeting the requirements of the MPDT. As shown in Fig. 3c, the maximum error rate of the magnetic field measured is 1.6%. Considering the position of the magnet and measuring error, the manufacturing accuracy of the superconducting magnet is strong and relatively uniform magnetic field is in favour of plasma ejection along the axis. Figure 3d shows the schematic diagram of superconducting MPDT.

### Numerical simulation of plasma magnetic nozzle effect

**Figure 4:**
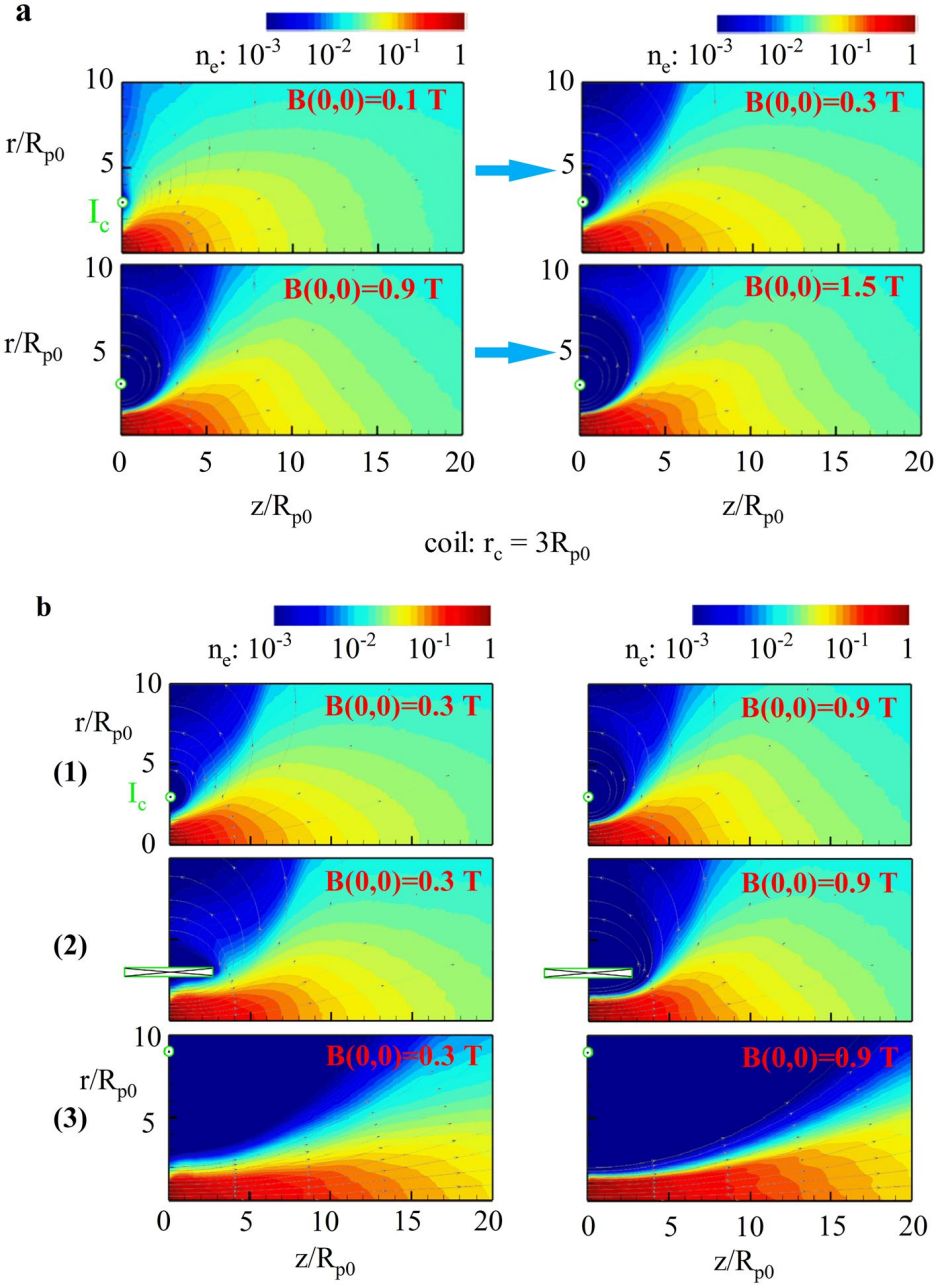
(**a**) The density distribution of the plasma number when the radius of the current ring was r_c_ = 3R_p0_ (initial plasma radius) and the central magnetic field was B (0, 0) = 0.1 T, 0.3 T, 0.9 T and 1.5 T. (**b**) Three magnetic field coil configurations and plasma number density distributions under different magnetic field strengths: (1) current loop radius r_c_ = 3R_p0_; (2) coil radius r_c_ = 3R_p0_, width H_c_ = 6R_p0_; (3) current loop radius r_c_ = 9R_p0_.

A two-dimensional plane model based on the PIC method^30^ was used to simulate the effects of magnetic fields with different configurations and field strengths on plasma plume acceleration. It is difficult to directly simulate the plasma discharge and the four acceleration modes, so we ignore the discharge process and only consider the movement trend of the ionized plasma in different magnetic field configurations, so as to guide us to choose a more appropriate applied magnetic field configurations. The magnetic field midpoint of the plasma flow Mach number was set to 1. The default magnetic field was produced by the single current, and under the condition of this specific field configuration, the plasma density distribution for different magnetic field intensities (as shown in Fig. 4a) was captured. Here, *r* represents the radius of the ring current or solenoid coil radius; *z* is axial direction length of coil; R_p0_ is plasma initial radius; r_c_ is the radius of the ring current; H_c_ is the width of solenoid coil; and B (0, 0) is the central magnetic field. The dimensionless method is used in the simulation, which can not only capture the main physical process, but also effectively reduce the computational effort. Specifically, the central magnetic field increased to B (0, 0) = 0.3 T, the trend of the plasma density distribution exhibited apparent deflection, and the plume expansion angle decreased relative to the lower magnetic fields. As the magnetic field increased further to B (0, 0) = 1.5 T, a portion of the plasma rotated along the magnetic lines. This indicates that, under the default magnetic-field configuration condition, increasing the magnetic field offers the advantage of reducing the angle of expansion of the plasma plume. However, an excessively strong magnetic field poses strong magnetic constraint on the plasma, which may lead to an increase in the expansion angle of the plasma plume and plasma reflux as shown at the bottom right in Fig. 4a. It can be predicted that the strong magnetic field means that the Lorentz force is larger and the deflection radius is smaller, so that part of the plasma spatters on the anode and other parts without leaving the acceleration zone, which leads to the failure of the MPDT to work normally and serious performance degradation.

With the increase in magnetic-field strength, the axial guiding effect of the magnetic field on the expansion trend of the plasma was more significant. By comparing the simulation results of the three potential magnetic configurations, as shown in Fig. 4b, it can be seen that the flattened axial magnetic field is favourable for the decrease in expansion angle of the plasma plume and for axial acceleration. Moreover, the reverse tendency of the plasma also decreased under certain strong magnetic field constraints, which supports the notion that a slim magnetic field configuration is conducive to the improvement of thrust performance.

### Performance test of the MPDT

**Figure 5:**
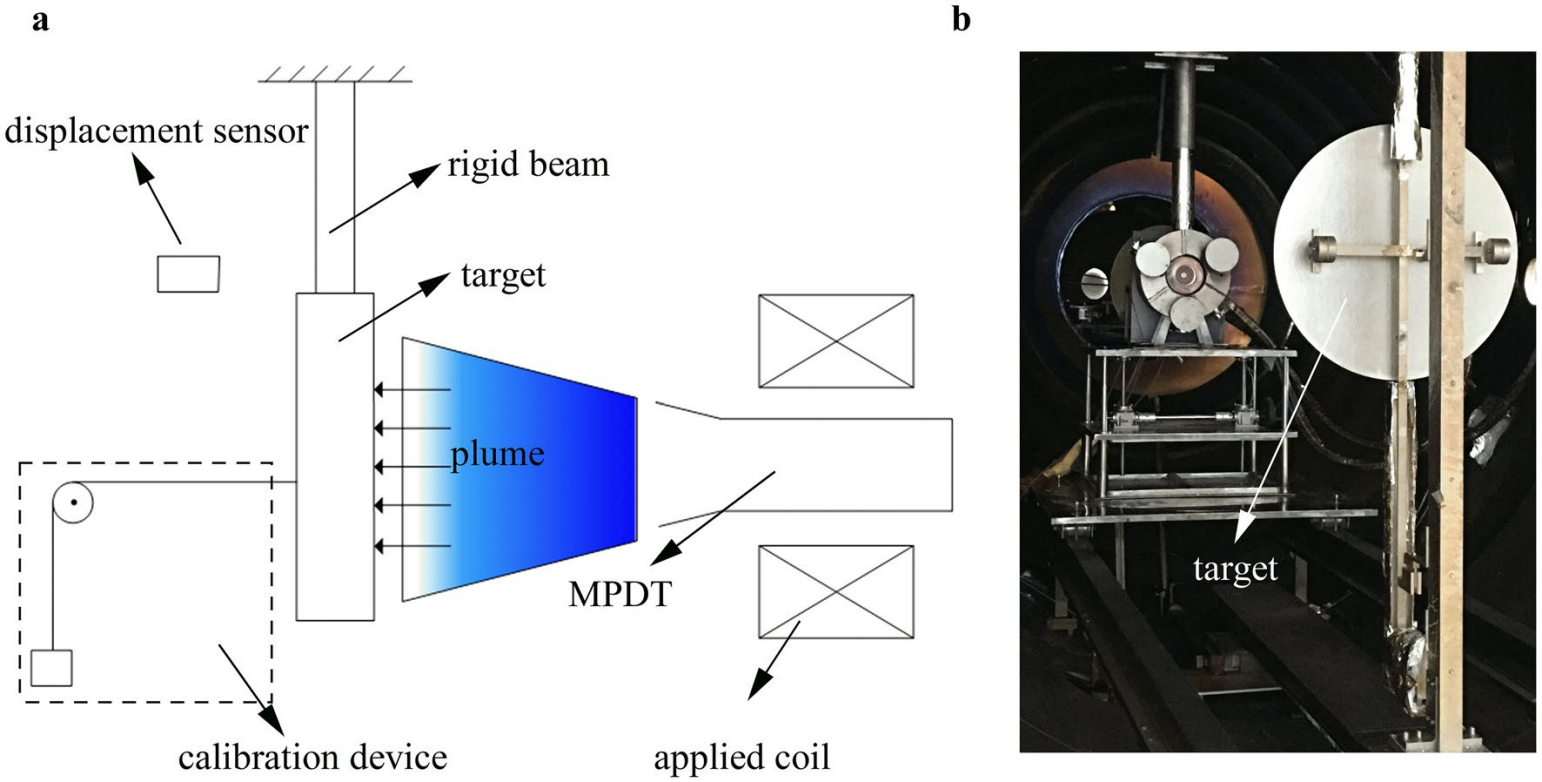
(**a**) Schematic diagram of target thrust measurement device, which can be calibrated using standard weights. (**b**) Photograph of the target thrust measurement device.

Thrust is an important parameter in MPDT performance testing. Directly measuring the thrust of an MPDT is technically very difficult due to its cumbersome power supply and feeding systems. The target measurement method is an indirect method that has been implemented and improved continually by our research team^31^. Its schematic diagram is shown in Fig. 5a. A target thrust measurement device is mainly composed of a rigid beam, target and displacement sensor. The target is fixed at the bottom of the rigid beam. It intercepts the plume so that thrust is transferred to the target surface, causing the beam to bend. Bending of the beam is measured by the displacement sensor, and the thrust of the plume on the target can be obtained by combining the calibration results of the thrust measurement device. The specific calibration method is as follows: A series of standard forces are applied to the target using weights, and the output voltages of the sensor are recorded to achieve calibration of the thrust measurement device. In the experiment, the actual thrust acting on the target surface was calculated from the output voltage of the sensor. A photograph of the target thrust measurement device is shown in Fig. 5b. The thrust of the calibrated Hall thruster was measured with the target thrust measurement device. The results show that the thrust measurement error is less than 1%, which is within the acceptable range of engineering measurement. And the calibration results show that the repeatability of target thrust measurement device can be more than 99.9% after calibration.

**Figure 6:**
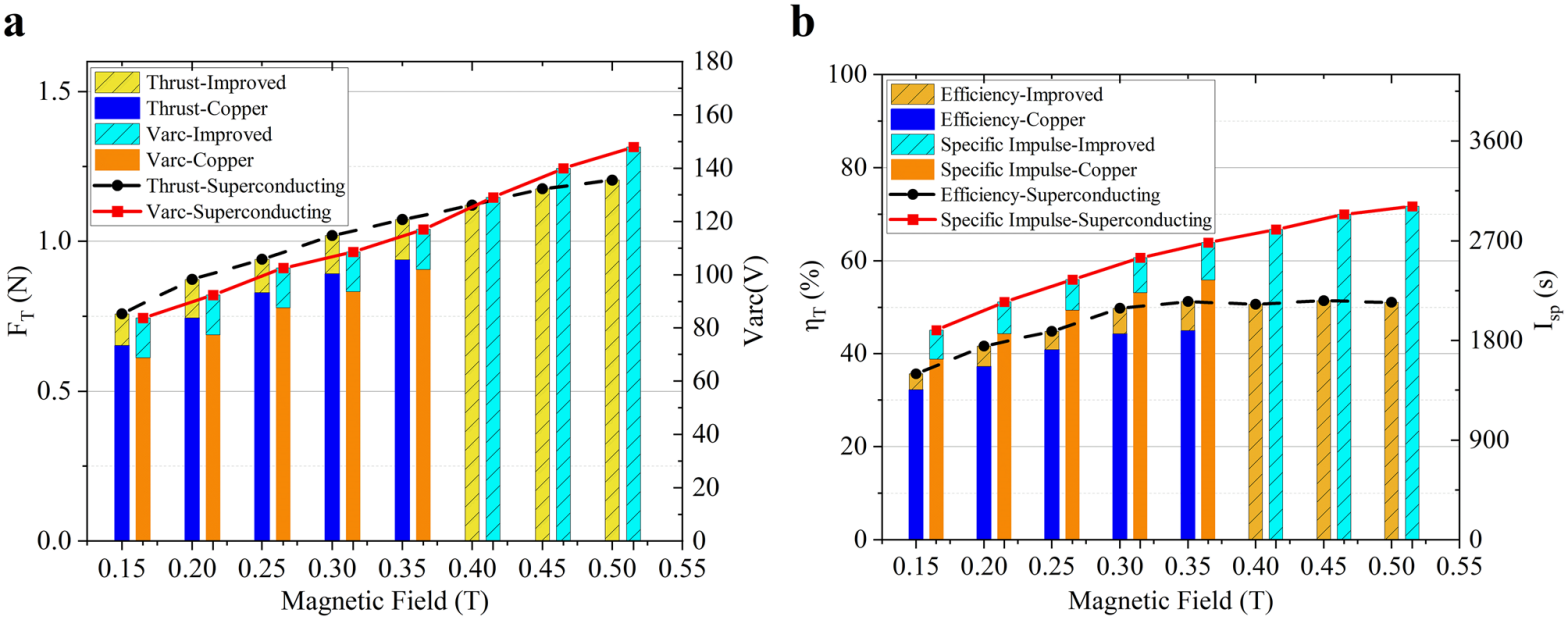
(**a**) Arc voltage V_arc_ and thrust F_T_ under different magnetic fields were compared between the copper and superconducting MPDT (serious plume refluxing occurred under the condition of high power (> 100 kW) and strong magnetic field (> 0.35 T) for the thruster with the copper magnet). (**b**) Specific impulse I_sp_ and thrust efficiency η_T_ under different magnetic fields were compared between the copper and superconducting MPDT.

The performance difference of the MPDT with the conventional copper coil and the superconducting coil was investigated. Figure 6a and b show the arc voltage V_arc_, thrust F_T_, specific impulse I_sp_ and thrust efficiency of the thruster η_T_ under different magnetic field intensity B_AF_, from 0.15 T to 0.35 and 0.5 T. The thrust-improved represents the specific thrust value of the MPDT with superconducting coil compared with that with copper coil. The discharge current I_d_ was 240 A and the mass flow rate of argon m_Ar_ was 40 mg/s. It can be seen that V_arc_, I_sp_ and η_T_ of the thruster increased with the enhancement of B_AF_, but the measured values of the MPDT with the superconducting coil were found to be larger than those with the copper coil. The thrust *F*_*Tcu*_ of the MPDT with the copper coil can be expressed using Eq. (7), where *K*_*cu*_ = 1.4656 N/T and *M* = 0.4465 N. Thrust *F*_*Tsup*_ of the superconducting MPDT can be expressed using Eq. (8), where K* = 0.8556 N/T and M* = 0.2330 N.

7 $$F_{Tcu} = K_{cu} B_{AF} + M$$

8 $$F_{Tsup} = K^{*}_{ } F_{Tcu} + M^{*}$$

As can be seen within the scope of a given magnetic field under certain conditions, the slope of formula *K* can be reduced but the intercept *M* grows. Furthermore, the superconducting magnetic field can improve the lower limit of the thrust value but growth with the magnetic field slows, indirectly confirming that the homogeneous field of the superconducting magnet promotes the ionisation of the propellant. This, therefore, improves the discharge voltage and ionisation rate of the propellant and, in turn, the contribution of the thrust-plasma quantity. The improvement to the magnetic field is conducive to the improvement of which in turn more efficiently converts the circumferential kinetic energy of ionised high-temperature plasma into axial kinetic energy^32^.

**Figure 7:**
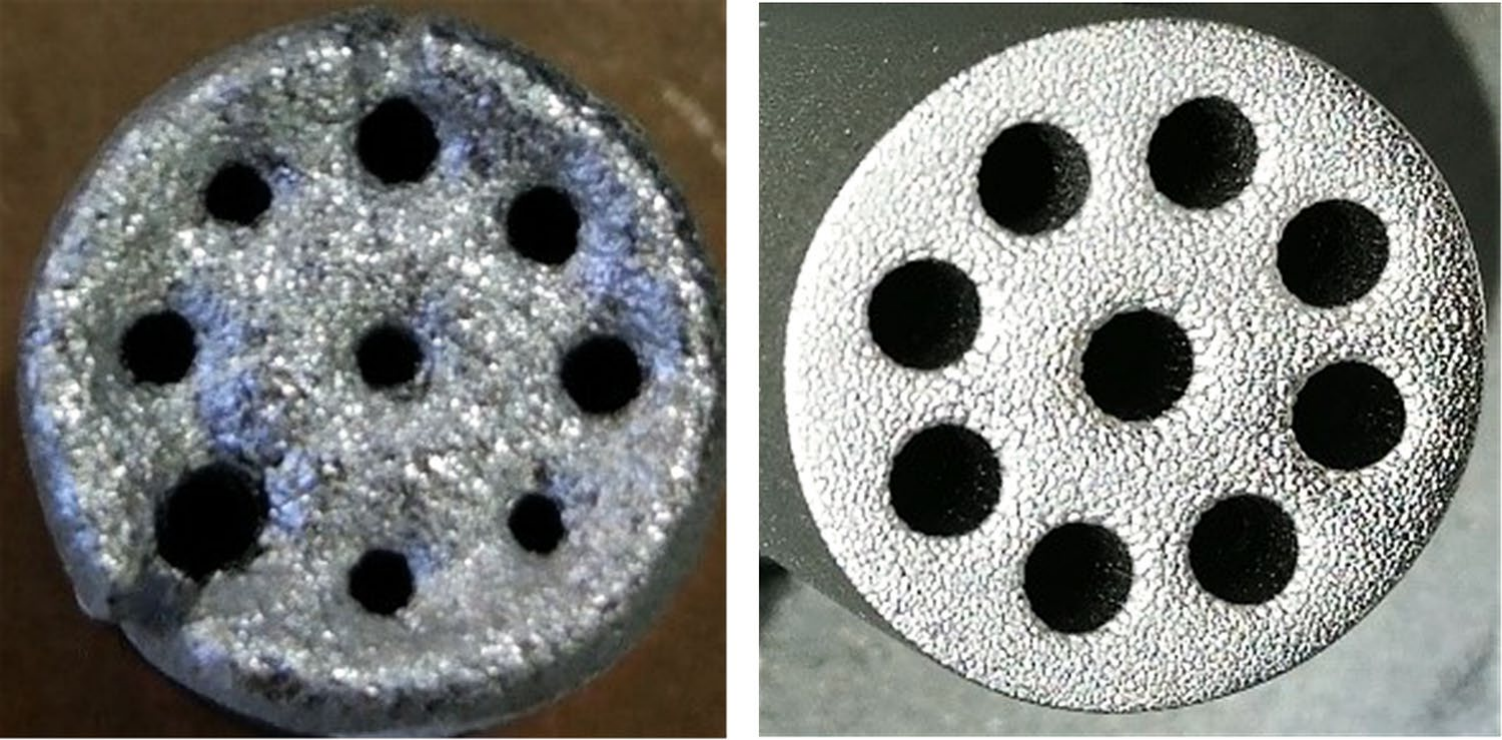
Comparison of ablation of cathodes under copper- (left) and superconducting-magnet conditions (right). Two cathode states under the same test conditions: discharge current of I_d_ = 240 A and discharge time of t_dis_ = 50 h.

Moreover, the superconducting MPDT showed superior performance due to the improved symmetry and axial magnetic field uniformity of the superconducting magnet. As shown in Fig. 7, the ablative comparison was made between the two cathode states under the same test condition, with I_d_ = 240 A and discharge time t_dis_ = 50 h. It can be seen that the cathode point used in the conventional copper magnet following ablation appeared to melt, and the sputtering on the surface was not uniform. In contrast, the cathode used in the superconducting magnet under the same test condition did not appear to melt and only a slight sputtering on the surface was observed. The highly homogeneous magnetic field of the superconducting magnet was capable of delaying the plasma cloud expansion rate in the hole of multi-cathode, causing radial compression of the high-temperature plasma cloud and moving predominantly downstream in the axial direction, thereby reducing ablation in the inner hole of cathode. Furthermore, the ions and temperature environment of the cathode point was improved, which in turn reduced the corrosion on the surface of the cathode and, thus, increased its service lifespan. Thruster cathode with superconducting coil has a service life of about 4000 h, and that with copper coil has a service life of about 2000 h under the same test conditions.

9 $$F_{Tsup2} = K_{2}^{*} F_{Tsup} + M_{2}^{*}$$

**Figure 8:**
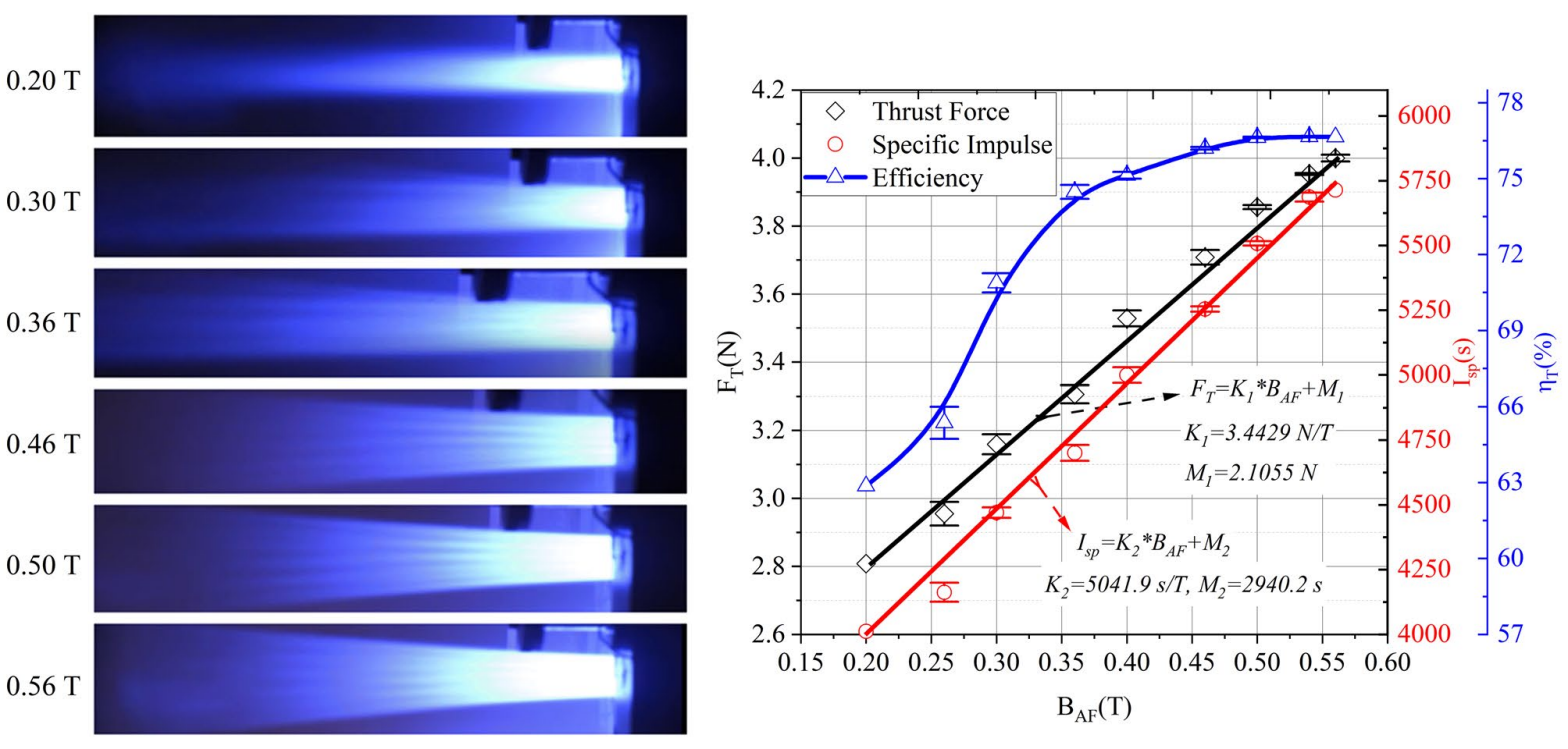
Performance of the superconducting MPDT. Changes of F_T_, I_sp_ and η_T_ to the superconducting MPDT under different magnetic fields, and the plume comparison with the magnetic field intensity of the superconducting magnet, from 0.20 to 0.56 T for the discharge current (I_d2_ = 700 A) and propellant argon mass flow rate (m_Ar2_ = 70 mg/s), respectively. Thrust and specific impulse are approximate to a straight line *F*_*T*_ = *K*_1_*B_*AF*_ + *M*_*1*_ and *I*_*sp*_ = *K*_2_*B_*AF*_ + *M*_*2*_ respectively (*K*_*1*_ = 3.4429 N/T, *M*_*1*_ = 2.1055 N, *K*_*2*_ = 5041.9 s/T and *M*_*2*_ = 2940.2 s).

Due to the serious plume refluxing of the MPDT under a condition of high power (> 100 kW) and the presence of a strong magnetic field (> 0.35 T) of the copper magnet for the inhomogeneity of its magnetic field, a performance test for the MPDT with the superconducting magnet was conducted. The performance parameters for the superconducting MPDT under different magnetic fields are shown in Fig. 8. Furthermore, the thrust *F*_*Tsup2*_ at I_d2_ = 700 A and m_Ar2_ = 70 mg/s can be expressed using Eq. (9), where *K*_2_* = 2.7615 N/T and *M*_2_* = 0.4191 N. The effect of the discharge current on the thrust lift was adjusted via M2*. Under certain conditions, the increase of I_d_ was found to be conducive to the promotion of the thrust, but the increase was smaller than the direct ratio. Similarly, under the superconducting magnetic field, higher performance parameters of the thruster could be achieved, where F_T_ = 4 N, I_sp_ = 5714 s, η_T_ = 76.6% at approximately 150 kW.

Figure 8 also shows the plumes under different magnetic fields. The superconducting MPDT experienced an obvious layered distribution at the plume flow path, which matched the porous cathode configuration. Moreover, with the increase in magnetic-field intensity, the plume flow became brighter and more concentrated on the cathode multi-channel, indicating that the ionisation efficiency of the propellant was higher. Under these conditions, when B_AF_ > 0.50 T, η_T_ remains almost constant, which means that increasing B_AF_ to increase η_T_ does not work in this case.

## Discussion

In this study, we designed a novel superconducting MPDT at the 150 kW level. With greatly reduced size and volume compared with the traditional copper coil, the superconducting magnet used in the MPDT helped to achieve improved plasma confinement capability based on a higher magnetic field strength. The optimization analysis showed that the thin and long magnetic field of the superconducting magnet exhibited superior homogeneity and played a significant role in improving the performance of the MPDT. Using the PIC method of microscopic particle simulation, the plasma magnetic nozzle effect and performance of the MPDT under different magnetic-field conditions were carefully studied. The empirical formula was obtained through the integrated experiment used, which showed that the thrust increased with increases in the magnetic field within a certain range, which is consistent with previous research^30^. Compared with the conventional copper coil thruster, the performance of the superconducting MPDT was improved. However, due to plume refluxing related to the thruster when the magnetic field is higher than 0.56 T, the performance of the superconducting MPDT at a stronger magnetic field was not investigated. In the future, the performance of the superconducting MPDT could be improved further by optimising the structure of the cathode and anode, improving the performance of the power supply and solving the discharge problem caused by plasma reflux. Moreover, the performance of the superconducting MPDT could be improved further by virtue of the high uniformity and strong magnetic field of the superconducting magnet.

## Method

### Experiment setup

Figure 2 shows the experimental setup and Fig. 5 shows the target thrust measurement device. A superconducting MPDT inside a cylindrical vacuum chamber (length = 6 m, radius = 1.5 m), with a vacuum degree of 10^–4^ Pa to simulate the space environment. The vacuum chamber was sufficiently large to prevent plasma sputtering from affecting the performance test of the thruster. The vacuum system can achieve the working vacuum degrees of about 0.1 Pa and 0.7 Pa respectively, when the propellant mass flow rates are 40 mg/s and 70 mg/s. The superconducting MPDT consisted predominantly of a cathode, anode and superconducting coil, as shown in Fig. 2. The anode and cathode were cooled via the water-cooling system during the operation. Argon gas was adopted as the propellant and had a maximum flow rate of 200 mg/s.

### Superconducting technology plasma confinement

A completely new way of using superconducting magnet technology to confine plasma with high energy and extremely high temperatures is proposed. Plasma is produced by ionizing the propellant and is confined by magnetic field so it can be accelerated and ejected. The coefficients of the formulations for thrust force and specific impulse are corrected based on the experimental results. The previous empirical formula was not based on high-magnetic-field test data. An improved PIC method related to the plasma magnetic nozzle effect was used to verify the plasma-number distribution under different field configurations.

### Systematic experiment validation

The superconducting magnet was integrated with the MPDT system to conduct a systematic experiment. To adapt the independent operating environment of the MPDT vacuum chamber, the superconducting magnet was cooled with a cryogenic system with no evaporation and low heat leakage. A simulated electromagnetic distribution with a magnetic-field configuration was used to determine the optimal superconducting magnet structure of the MPDT. A new systematic observation platform was established to monitor the system status (including thrust, specific impulse, charge current, superconducting magnet operating parameters). The key parameter of thruster, thrust is measured by target measuring device and then obtained by voltage signal conversion, based on thrust balance principle. Differences in the performance of the MPDT under two conditions—conventional copper coil versus superconducting coil—and different magnetic-field intensities, were experimentally investigated.

## Data Availability

The data that support the findings of this study are available from the corresponding author upon reasonable request.
