# Author Correction: Integrated study on the comprehensive magnetic-field configuration performance in the 150 kW superconducting magnetoplasmadynamic thruster

**DOI:** 10.1038/s41598-021-01675-8

**Published:** 2021-11-10

**Authors:** Jinxing Zheng, Haiyang Liu, Yuntao Song, Cheng Zhou, Yong Li, Ming Li, Haibin Tang, Ge Wang, Yuntian Cong, Baojun Wang, Yibai Wang, Peng Wu, Timing Qu, Xiaoliang Zhu, Lei Zhu, Fei Liu, Yuan Cheng, Boqiang Zhao

**Affiliations:** 1 Institute of Plasma Physics, Hefei Institutes of Physical Science, Chinese Academy of Sciences, Hefei, 230031 China; 2 University of Science and Technology of China, Hefei, 230026 China; 3 Beijing Institute of Control Engineering, Beijing, 100080 China; 4 Beihang University, Beijing, 100191 China; 5 Tsinghua University, Beijing, 100084 China

Correction to: *Scientific Reports* 10.1038/s41598-021-00308-4, published online 19 October 2021

In the original version of this Article, authors Jinxing Zheng and Yuntao Song were incorrectly affiliated with ‘University of Science and Technology of China, Hefei, 230026, China’. The correct affiliation for both authors is listed below.

Institute of Plasma Physics, Hefei Institutes of Physical Science, Chinese Academy of Sciences, Hefei, 230031, China

The original Article has been corrected.

